# Computational prediction of heterogeneous interface properties at the atomic level

**DOI:** 10.1186/1758-2946-5-S1-O10

**Published:** 2013-03-22

**Authors:** Lucio Colombi Ciacchi

**Affiliations:** 1 Hybrid Materials Interfaces Group, Faculty of Production Engineering and Bremen Centre for Computational Materials Science, University of Bremen, 28213 Bremen, Germany

## 

The interactions across interfaces between different phases govern the behavior of a wide range of systems of high technological importance, such as catalysts (gas/solid), energy storage (liquid/solid or solid/solid) or biomedicine (liquid/solid), just to cite a few examples. The simulation of such systems poses considerable challenges due to their inherent complexity, which often cannot be captured by idealized models containing only a few tens of atoms. However, large system sizes can only be modeled by means of approximate methods using force field potentials, whose predictive power and transferability can be questionable if a direct comparison of the simulation results with experimental observables is sought. Moreover, efficient sampling of the phase-space is strictly required to computationally predict measurable quantities such as adsorption free energies or adhesion forces. In this talk, I will present a strategy for the development of realistic models of oxidized metals and of force field potentials that capture the essential interactions at oxide/water interfaces, based on extensive DFT simulations. Our potentials can be seamlessly combined with widely used bimolecular force fields and predict free energy of adsorption and adhesion forces of polypeptides in quantitative agreement with experiments. This has been achieved by means of advanced molecular dynamics simulations combining Metadynamics with Replica Exchange with Solute Tempering methods, which enable converged calculations of free energies for such complex systems [1]. Our simulations reveal that polypeptides are able to sense the local variation of the water density at the interface in a way that resembles the "lock-and-key" bimolecular recognition. I will also present results of atomistic simulations of the contact forces between TiO_2 _nanoparticles in aggregates under humid conditions. Coupled with AFM force spectroscopy experiments, the simulations reveal that the mechanisms underlying particle-particle adhesion comprise a combination of "continuum" capillary forces and "discrete" contributions arising from the structuring of physisorbed water molecules at the oxide surfaces [2]. Preliminary results about the dependencies of the contact forces on particle size and humidity indicate the applicability limits of capillary theories in the sub-10-nm particle size range.
